# Glyceraldehyde as an Efficient Chemical Crosslinker Agent for the Formation of Chitosan Hydrogels

**DOI:** 10.3390/gels7040186

**Published:** 2021-10-28

**Authors:** Pierre Carmona, Anca M. Tasici, Sverre A. Sande, Kenneth D. Knudsen, Bo Nyström

**Affiliations:** 1 Department of Chemistry, University of Oslo, N-0315 Oslo, Norway; pierre.carmona@chalmers.se; 2 Department of Physics, Division of Nano-and BioPhysics, Chalmers University of Technology, Fysikgränd 3, 412 96 Gothenburg, Sweden; 3 Department of Pharmacy, Section for Pharmaceutics and Social Pharmacy, University of Oslo, N-0316 Oslo, Norway; ancamtasici90@gmail.com (A.M.T.); s.a.sande@farmasi.uio.no (S.A.S.); 4 Institute for Energy Technology, N-2027 Lillestrøm, Norway; Kenneth.knudsen@ife.no

**Keywords:** chitosan, glyceraldehyde, hydrogels, chemical crosslinking, rheology, SANS, gelation time, viscosity, postgel

## Abstract

The rheological changes that occur during the chemical gelation of semidilute solutions of chitosan in the presence of the low-toxicity agent glyceraldehyde (GCA) are presented and discussed in detail. The entanglement concentration for chitosan solutions was found to be approximately 0.2 wt.% and the rheological experiments were carried out on 1 wt.% chitosan solutions with various amounts of GCA at different temperatures (25 °C and 40 °C) and pH values (4.8 and 5.8). High crosslinker concentration, as well as elevated temperature and pH close to the pK_a_ value (pH ≈ 6.3–7) of chitosan are three parameters that all accelerate the gelation process. These conditions also promote a faster solid-like response of the gel-network in the post-gel region after long curing times. The mesh size of the gel-network after a very long (18 h) curing time was found to contract with increasing level of crosslinker addition and elevated temperature. The gelation of chitosan in the presence of other chemical crosslinker agents (glutaraldehyde and genipin) is discussed and a comparison with GCA is made. Small angle neutron scattering (SANS) results reveal structural changes between chitosan solutions, incipient gels, and mature gels.

## 1. Introduction

Hydrogels exemplify an appealing class of soft materials with specific functionalities, and they have emerged as three-dimensional matrices for biomedical applications, including regenerative medicine and drug delivery systems [1,2]. Hydrogels are physically or chemically crosslinked hydrophilic polymer chains forming a three-dimensional network capable of absorbing large amounts of water. One important member of this class of gel-forming materials is chitosan, a linear copolymer of β-(1-4)-linked 2-acetamido-2-deoxy-D-glucopyranose and 2-amino-2-deoxy-D-glucopyranose, generally obtained by alkaline deacetylation from marine chitin [3,4]. In contrast to many other polysaccharides, chitosan dissolved in acid aqueous media is positively charged because of protonation (the degree of protonation depends on the pH of the medium) of primary amines on the chitosan chains, which give the polymer a polyelectrolyte character. Chitosan exhibits many favorable biomedical characteristics, such as biodegradability, nontoxicity, and biocompatibility [5].

Different approaches have been employed to prepare chemically crosslinked chitosan hydrogels. The most common chemical crosslinker agents include *N*,*N*′-methylenebisacrylamide [6], glutaraldehyde [7], genipin [8], formaldehyde [9], ethylene glycol diglycidyl ether, epichlorohydrin [10], and aldehyde-terminal benzoxazine [11]. Most of these chemicals, except genipin, are cytotoxic and they are not appropriate for making gels to be used in biomedical applications. Genipin is a biocompatible compound that has been considered for pharmaceutical and medical gel-applications [12,13]. In spite of the frequent use of genipin to form chitosan hydrogels, we are not aware of systematic rheological studies monitoring the formation of chitosan macroscopic hydrogels with this crosslinker. 

The addition of genipin to chitosan leads to the formation of crosslinks between primary amine groups and a crosslinked network evolves [14,15]. However, it has been observed [14] that the crosslinking process of chitosan with genipin is complicated by the oxygen radical-induced polymerization of genipin that takes place as the heterocyclic genipin compound quickly linked to chitosan. This process caused the formed gel to assume a blue color in the presence of air. The blue coloration was initially found to be more marked at the interface of the gelled sample but gradually moved down through the sample with time. To avoid these complications, we decided to utilize glyceraldehyde (GCA), which is another biocompatible crosslinker agent-forming gel that is easy to reproduce and characterize. The chemical crosslinking agents are usually divided into two different categories referred to as zero-length and non-zero-length crosslinkers. GCA belongs to non-zero-length crosslinkers and for chitosan this type of crosslinker is incorporated into the crosslinked network structure, whereas a typical zero-length crosslinker like 1-ethyl-3-(3 dimethylamino propyl) carbodiimide hydrochloride (EDC) is not built into the crosslinked gel matrix.

GCA can covalently crosslink primary amino acid groups residing on biopolymers, such as chitosan, to form hydrogels [16]. Genipin is usually considered to be less cytotoxic than other common crosslinker agents used for biopolymers containing residues with primary amine groups. However, in a recent cytotoxic study [17] of various crosslinker agents on the cytotoxicity of four different cell lines it was found that GCA is less cytotoxic than genipin. The hypothesis is that GCA can be utilized as an efficient crosslinker agent for chitosan to form macroscopic hydrogels that can be systematically characterized by rheological methods during the gelation process. 

In the past, GCA has mostly been utilized for the crosslinking of different proteins [18,19,20]. To the best of our knowledge, there is no reported study where GCA has been employed to crosslink chitosan to form macroscopic hydrogels. It has only been utilized in the formation of microparticles [21].

The aim of this work is to present a systematic characterization of the rheological and structural features during the gelation process of chitosan in the presence of GCA. Chitosan hydrogels are utilized for various biomedical applications, such as scaffolds in tissue engineering, and for this purpose it is important to control the gelation features and to understand how external parameters like temperature and pH influence the gelation ability and how the different conditions affect the formation of incipient and mature gels. In view of this, the effects of crosslinker concentration, temperature, and pH on the rheological features during the gelation process are investigated. In this way, we hope to gain a fundamental insight into the factors that govern the properties of both the incipient and the long-cured gel. Characteristic assets, such as the gel point, gel strength, and structure of incipient gels are determined. In addition, the evolution of the solid-like response of long-cured gels is monitored and the mesh size of long-cured gels is determined at different conditions of crosslinker concentration and temperature. To gain insight into differences in the behavior of diverse crosslinker agents in the gelation of chitosan, we have made a simple comparison of the gelation process by using GCA, glutaraldehyde (GTA), or genipin (GP). In addition, we conducted small angle neutron scattering (SANS) experiments during the gelation of chitosan in the presence of GP to elucidate how the local structure of the network is affected in the course of gelation to long-cured gels.

## 2. Results and Discussion

### 2.1. Shear Viscosity Measurements and Entanglements

Before the results from the gelling systems are presented and discussed, the choice of the chitosan concentration that is utilized in this work will be debated. To form a chemically crosslinked macroscopic gel from a polymer solution, the concentration of the polymer must be in the semidilute regime [22]. The commencement of this regime can be estimated from the overlap concentration c* = *γ*/[*η*], where the constant *γ* = 1 and [*η*] is the intrinsic viscosity. This provides a simple definition [23,24] of c* that is widely accepted for demarking the transition from the dilute to the semidilute concentration regime. From viscosity data from a capillary viscometer, the overlap concentration was estimated to be c* ≅ 0.02 wt.% for the chitosan samples. From a viscosity study [25] of chitosan solutions, values of *γ* in the range 0.5–2 were reported. In this region, the polymer chains overlap each other and form a transient network [22]. In a previous study [26] on polymer concentration-induced chitosan gels, it was shown that entanglements are significantly more efficient to produce high gel strength of incipient gels than hydrophobic interactions. In view of this, it is argued that to be able to prepare gels of high mechanical strength with potential for tissue engineering, it is needed to crosslink a chitosan solution that is sufficiently concentrated to be in the entangled regime. It has been argued [27,28] that entanglements may play a role in the elastic response in gels. To understand how the entanglement situation is influenced by different conditions of temperature and pH, as explored in this work, the concentration dependence of the zero-shear specific viscosity in both the unentangled and entangled concentration regime is investigated. 

Shear viscosity measurements on polymer solutions have the potential to reveal the crossover from unentangled to entangled conditions [24]. For this purpose, the zero-shear viscosity for chitosan solutions of different concentrations (Supplementary Materials Figure S1) must be determined. At low concentrations, Newtonian behavior [24] is observed at all shear rates, whereas for the higher concentrations (entangled solutions) shear thinning is evident at higher shear rates as the network becomes disrupted.

**Figure 1:**
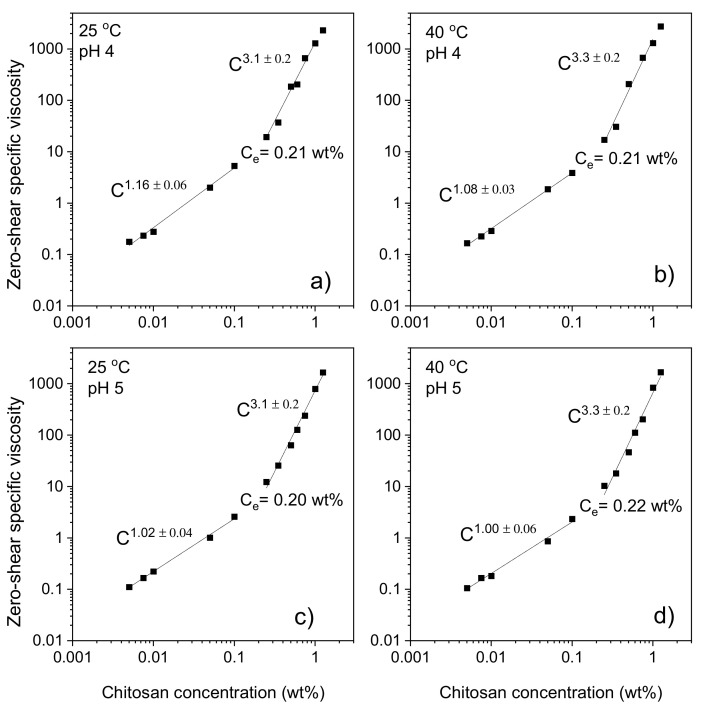
Log–log plot of the concentration dependence of the zero-shear specific viscosity for chitosan solutions at different temperatures and pH values indicated. (**a**) pH 4 and 25 °C, (**b**) pH 4 and 40 °C, (**c**) pH 5 and 25 °C, (**d**) pH 5 and 40 °C. The errors in the power law exponents are standard deviations.

Figure 1 shows log–log representations of the concentration dependences of the zero-shear specific viscosity $\eta_{sp}^{0}$ ($\eta_{sp}^{0}$ ≡ ($\eta_{sol}^{0}$/$\eta_{solv}^{0}$) − 1), where $\eta_{sol}^{0}$ is the zero-shear viscosity of the solution and $\eta_{solv}^{0}$ is the viscosity of the solvent) at different temperatures and pH values in chitosan solutions without any added crosslinker agent. In all cases, the entanglement concentration *c_e_* is roughly 0.2 wt.%, which is approximately ten times larger than the estimated overlap concentration c*. The entanglement concentration is virtually unaffected by the considered temperatures and pH values. It is known that temperature may affect the strength of hydrogen bonds and hydrophobic interactions [29,30], but this does not seem to influence the value of the crossover concentration. This suggests that the chain entanglement interactions are not significantly affected by the changes in temperature and pH. At pH values below pK_a_ (pH ≈ 6.3–7) for chitosan, the number of protonated amino groups increases and the charge density and the polyelectrolyte effect is enhanced, but it is possible that a pH change from 4 to 5 is too little to affect the charge density.

Changes of pH in chitosan solutions will lead to alteration of the charge density of the polymer; thereby modifying the polyelectrolyte characteristics. It is interesting to note that, in rheological studies [31,32] of aqueous solutions of sodium carboxymethyl cellulose, no effects of salt addition on the entanglement concentration and entanglement density were reported. This advocates that the density of binary contacts in solution, or topological constraints, should not be affected by the ionic strength. 

The concentration dependences of $\eta_{sp}^{0}$ in the unentangled semidilute concentration regime of nonionic polymers can theoretically be described in the framework of the Rouse model and the scaling approach [22,33]:

(1) $$\eta_{sp}^{0}\sim c^{1/\left(3\nu -1\right)}\sim \left\{\begin{array}{c} c^{2}\;\left(\nu =0.5,\;theta\;solvent\;conditions\right)\\ c^{1.30}\;\left(\nu =0.59,\;good\;solvent\;conditions\right) \end{array}\right.$$

where *ν* is the excluded volume exponent at theta and good solvent conditions, respectively. The scaling model, together with the reptation prediction yields the following expression for the entangled semidilute regime [22] $\eta_{sp}^{0}\sim c^{\frac{3}{3\nu -1}}\sim$*c*^3.9^ at good solvent conditions. From a straightforward scaling approach, we would then have an exponent of 6 at theta solvent conditions. However, the simple scaling law breaks down under theta solvent conditions [34,35,36,37]. This was ascribed to the existence of two length scales in semidilute solutions at theta solvent conditions [36]. Based on that framework, the following power law was derived [36]; $\eta_{sp}^{0}\sim$*c*^4.7^. When chitosan is dissolved in 1 wt.% acetic acid, the polymer may, depending on the pH, exhibit a polyelectrolyte character. In view of this, the scaling laws for salt-free semidilute polyelectrolyte solutions are given. In the unentangled regime, the Fuoss law $\eta_{sp}^{0}$~*c*^0.5^ predicts the behavior and in the entangled domain the power law is given by $\eta_{sp}^{0}$~*c*^1.5^ [37,38,39]. This reveals that the power law exponents for polyelectrolytes are much lower than for solutions of nonionic polymers.

In the region prior to the entanglement concentration, the concentration dependence of $\eta_{sp}^{0}$ is found to follow a power law $\eta_{sp}^{0}$ ~*c^α^*, where α is close to 1 for all systems (Figure 1). In the concentration range above c_e_, $\eta_{sp}^{0}$ can be described by another power law $\eta_{sp}^{0}$~*c*^β^ with values of *β* in the domain 3.1–3.3. The values of both α and *β* are significantly lower than the corresponding theoretical values (*α* = 1.3 and *β* = 3.9) at good solvent conditions; cf. discussion above. It is interesting to note that both the values of the entanglement concentration and the power law exponents are nearly unchanged as the temperature and pH are altered. These findings suggest that the entanglement situation in the chitosan solutions is only slightly affected by the pH and temperature changes. It is possible that the lower values of both *α* and *β* observed for the chitosan solutions, can be traced to a weak polyelectrolyte effect from chitosan. In a previous rheology study [25] on chitosan solutions at a high ionic strength (screening of electrostatic interactions), the values of α and *β* were found to be 1 and 5.2, respectively. The value of *β* is much higher than expected (*β* = 3.9) for entangled solutions at good solvent conditions; this may indicate that the thermodynamic conditions are deteriorated upon addition of the electrolyte, and this can lead to a higher value of β than the theoretical model [36] predicts (*β* = 4.7) at theta solvent conditions. Shear viscosity studies on aqueous solutions of several neutral polysaccharides, such as dextran (*α* = 1.4 and *β* = 3.8) [40], polysaccharide intercellular adhesion (*α* = 1.27 and *β* = 4.25) [41], hydroxyethyl cellulose (*α* = 1.45 and *β* = 4.21) [42], and hydroxypropyl cellulose (*α* = 1.5 and *β* = 4.2) [43] have shown values of the slopes in the range (*α* = 1.3–1.5) and (*β* = 3.8–4.3). Shear viscosity results have also been reported for ionic polysaccharides and lower values of *α* and *β* were found [37,44].

### 2.2. Gelation of Chitosan in the Presence of Glyceraldehyde

The crosslinking of chitosan chains in aqueous solutions is mediated mainly through interaction between carbonyl groups of DL glyceraldehyde (GCA) and free amine groups on chitosan; over time, this reaction leads to gelation. The crosslinking is part of the Maillard reaction, which encompasses a complex network of reactions taking place over time. In the spirit of the approach of Tessier et al. [16], some of the reaction paths are outlined in Supplementary Materials Figure S2 to illustrate the complexity of the gelation process. In the description of this illustration in the Supplementary Materials, the possible reaction paths are depicted and briefly discussed, but it is beyond the scope of this work to investigate the impact of the different paths on the overall crosslinking reaction. Although the details of the chemical reactions that can influence the kinetics of the crosslinking process will not be further discussed, the effect of pH on the rheological results (see the Discussion below) demonstrates that the number of free amino groups on the chitosan chains is crucial for the rate of the crosslinking reaction. Even if the specific stimulus of the other reaction paths on the reaction kinetics of the crosslinking reaction is not analyzed, the deprotonated amino groups play an important role for the crosslinking process.

**Figure 2:**
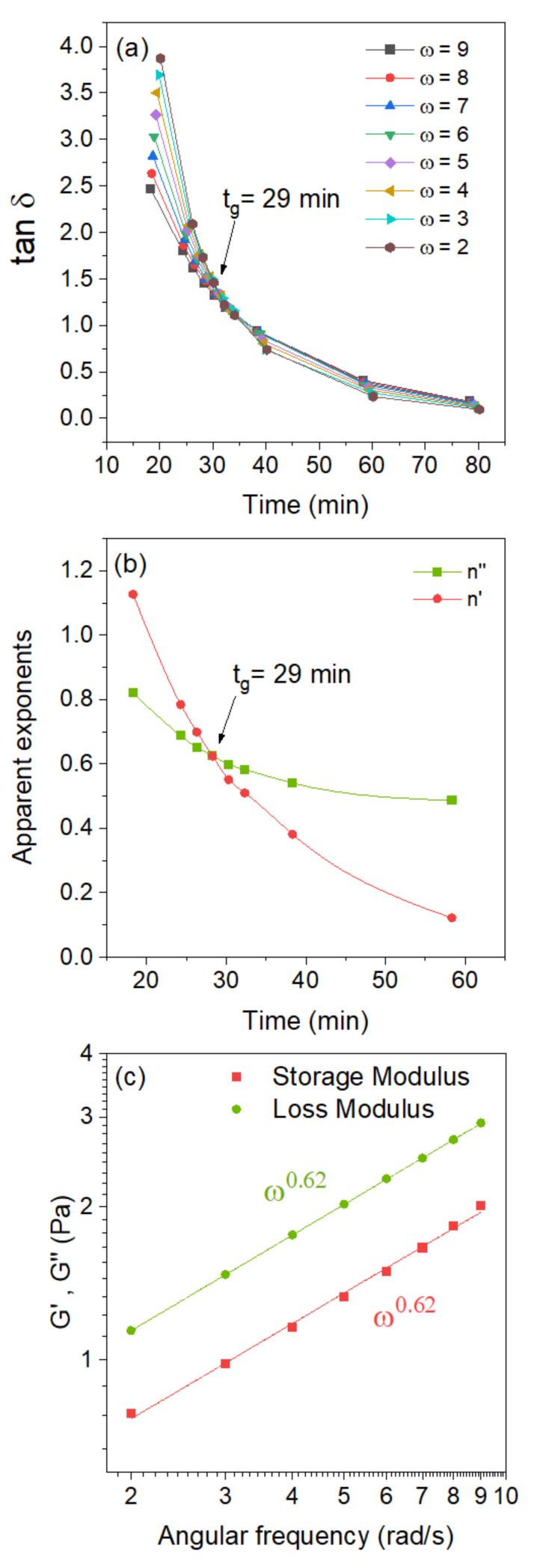
Determination of the gel point for 1 wt.% solutions of chitosan in the presence of glyceraldehyde (1 wt.%) at pH 5.8 and at a temperature of 40 °C. (**a**) Viscoelastic loss tangent as a function of time at the indicated angular frequencies (*ω*; rad/s). (**b**) Changes in the apparent relaxation exponents, *n*′ for the storage and *n*″ for the loss modulus, at various times and the intersection determining the gel point. (**c**) The power law behavior of the dynamic moduli at the gel point.

The incipient gelation and viscoelastic features of semidilute chitosan systems in the presence of the chemical crosslinker GCA can be monitored by using oscillatory sweep experiments. In the framework of a method developed by Winter et al. [45,46,47], the gelation time can be found through the observation of a frequency-independent value of tan *δ* (=*G*″/*G*′) (the phase angle between stress and strain) attained from a multi-frequency plot of tan δ versus time. Alternatively, the gel point can be established [48] by plotting the “apparent” viscoelastic exponents *n*′ and *n*″ (*G*′~*ω^n^*^′^, *G*″~*ω^n^*^″^), obtained from the frequency dependences of *G*′ and *G*″ at different times, and observing a crossover where *n*′ = *n*″ = *n*. At the gel point, the following power law is valid: *G*′~*G*″~*ω^n^* (0 < *n* < 1) and tan *δ* = tan (*n*π/2). These features are illustrated in Figure 2 for 1 wt.% chitosan solution in the presence of 1 wt.% glyceraldehyde at pH 5.8 and a temperature of 40 °C. Figure 2a shows a multi-frequency plot of tan *δ* versus time and the observation of a frequency-independent value of the loss tangent at the gel point. The crossover of the “apparent” viscoelastic exponents yields the same gel point as the previous method (Figure 2b). At the gel point, log–log plots of *G*′ and *G*″ versus angular frequency produce parallel lines as expected from the theoretical model (Figure 2c).

Based on the model described above, the gel strength of an incipient can be expressed in the following way [45]:

(2) $$G^{\prime }=\frac{G^{\prime \prime }}{tan\delta }=S\omega^{n}\Gamma \left(1-n\right)cos\delta$$

where Γ(1 − *n*) is the gamma function, *n* is the relaxation exponent, *δ* is the phase angle, and *S* is the gel strength parameter that depends on the crosslinking density and the molecular chain flexibility. 

Muthukumar [49] advanced a model, founded on the hypothesis that variations in the strand length between crosslinking points of the incipient gel network give rise to changes of the excluded volume interactions, to rationalize values of *n* in the completely accessible range (0 < *n* < 1). In the framework of this model, Muthukumar established a relationship between *n* and the fractal morphology of the incipient gel network through the expression

(3) $$\;n=\frac{d\left(d+2-2d_{f}\right)}{2\left(d+2-d_{f}\right)}$$

where *d* (*d* = 3) is the spatial dimension and *d_f_* is the fractal dimension that describes the relation between the mass of a molecular cluster in the network to its radius through the expression $R^{d_{f}}\sim M.$ For the gel network, larger values of *d_f_* suggest the evolution of a tighter network structure [47].

**Figure 3:**
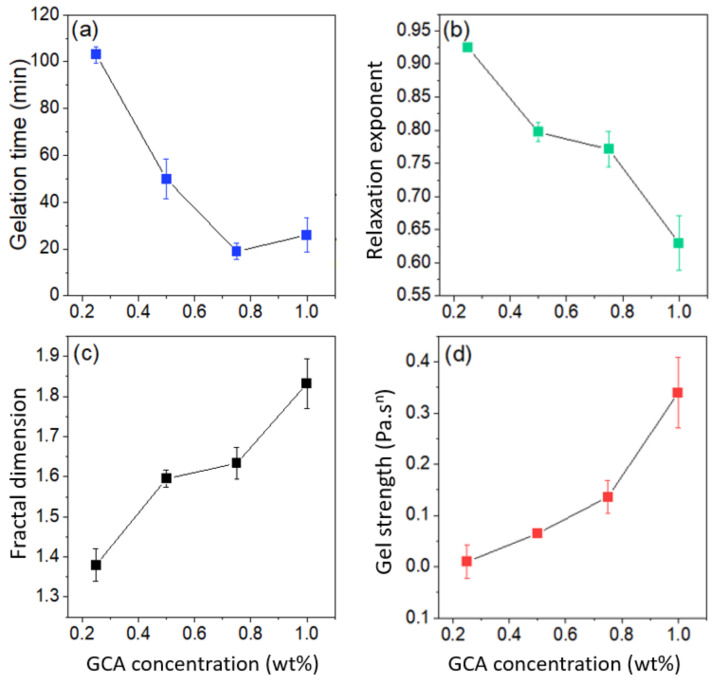
Effect of crosslinker concentration on (**a**) gelation time, (**b**) relaxation exponent, (**c**) fractal dimension, and (**d**) gel strength for 1 wt.% chitosan solutions at pH 5.8 and 40 °C. The error bars represent the standard deviation.

The effects of adding various amounts of crosslinker agent on the gelation time, relaxation exponent, fractal dimension, and gel strength are depicted in Figure 3. A transient network is formed at polymer concentrations above the crossover concentration in the semidilute regime; upon addition of a crosslinker agent, a permanent sample-spanning gel network evolves as a response to the crosslinking process. The gelation time decreases with increasing crosslinker concentration, because the probability of creating interchain crosslinks is enhanced with increasing crosslinker concentration (Figure 3a). However, at a sufficiently high crosslinker concentration, the solution is saturated with active crosslinking molecules and further increase in the added crosslinker agent will not considerably affect the gelation time (see Figure 3a). It is possible that the behavior at the highest crosslinker concentration is a sign of that the fast-crosslinking reaction path is suppressed by a slower reaction path among the paths outlined in Supplementary Materials Figure S2. Declining gelation time with increasing amount of crosslinker has been reported for various polymer-crosslinker pairs [50,51]. The value of the relaxation exponent is found to drop with increasing crosslinker concentration. A value of *n* = 0.5 was reported [46] for stoichiometrically balanced gels, *n* < 0.5 for gels with excess crosslinker agent, and *n* > 0.5 for gels with deficit crosslinker agent [45,52]. In light of this, the values of *n* observed in Figure 3b suggest that the crosslinker concentration is below that of a balanced gel. There are also studies [53,54,55] in the literature reporting values of *n* near to 0.7, which is close to the theoretical prediction, based on a percolation network (*n* = 0.72) [22,56], and the Rouse model with percolation statistics (*n* = 2/3) [53].

The fractal dimension increases (from Ca. 1.4 to 1.8) with increasing crosslinker concentration (Figure 3c) and this finding suggests the evolution of a critical gel with a “tighter” network structure [47,49,57]. This collaborates with the intuitive picture that a more extensive crosslinking process should lead to a more compact network [58,59]. As discussed below, this is also true for long-cured gels. In a previous study [60] on aqueous chitosan systems, concentration-induced gelation was monitored with rheometry and a fractal dimension of 2.2 was determined. In a more recent rheology investigation [61] on the concentration-induced gelation of chitosan-phosphoric acid and chitosan-oxalic acid systems, a fractal dimension of 1.9 was found for both systems. For the concentration-induced gels, the polymer concentration is relatively high (4–5 wt.%) and this leads to tight gel networks and high fractal dimensions. For chemically crosslinked gel networks, the tightness of the network depends on the crosslinker concentration. The strength of the gel depends on the crosslinking density and the gel strength increases with increasing crosslinker concentration, as depicted in Figure 3d. This type of behavior has been reported also for other types of chemically crosslinked gels [50,51,62,63]. 

To monitor the evolution of the viscoelasticity during the gelation process from the pre-gel to the post-gel regime, it is advantageous to introduce the complex viscosity in terms of its absolute value |η*(*ω*)*|* given by [24]

(4) $$\left|\eta *(\omega )\right|={\left(G^{’2}+G’{’}^{2}\right)}^{1/2}/\omega$$

In an analogous way, as for the dynamic moduli, the frequency dependence of the absolute value of the complex viscosity can be written [52] in the form of a power law |η*(*ω*)*|*~*ω^m^*, where the exponent *m* is related to *n* through the relation *m* = *n* − 1. Values of *m* close to zero signal liquid-like behavior, whereas values of m approaching −1 suggest a solid-like response.

**Figure 4:**
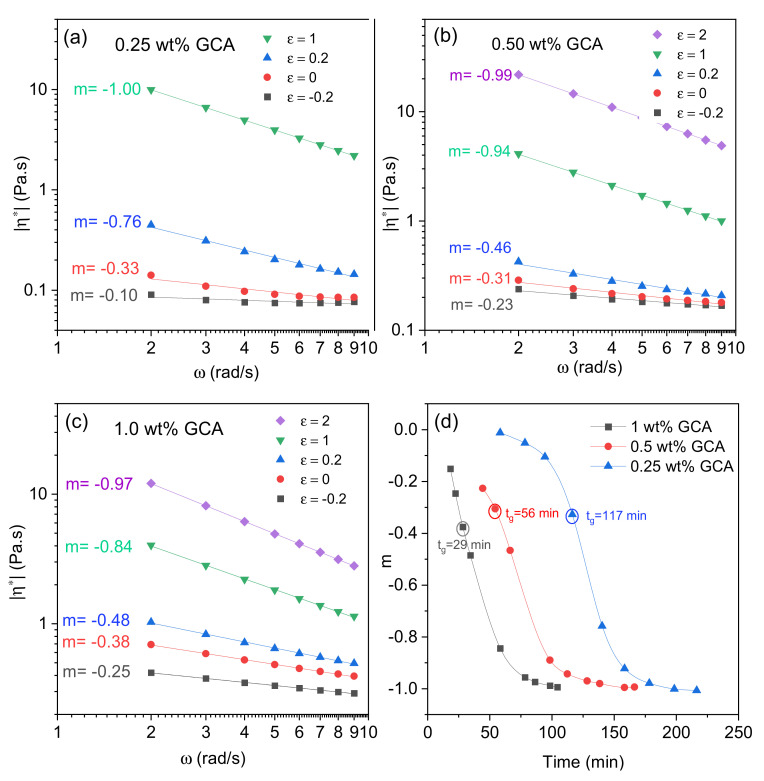
(**a**–**c**) Frequency dependence of the absolute value of the complex viscosity (log-log plot) in the course of the gelling process at different stages (*ε*) for 1 wt.% chitosan sample at a temperature of 40 °C and pH = 5.8 and at the crosslinker (GCA) concentrations indicated. (**d**) Plot of the power law exponent m versus time at the crosslinker concentrations and gel points indicated.

In Figure 4a–c, the frequency dependencies of the absolute value of the complex viscosity are depicted at various stages (where *ε* = (*t* − *t_GP_*)/*t_GP_* is the relative distance to the gel point (GP)) in the course of the gelation process of chitosan samples with different crosslinker concentrations. In the pre-gel region (*ε* < 0) a weak frequency dependence of |η*(*ω*)*|* is observed for all systems and the low values of m suggest liquid-like behavior, whereas at long times in the post-gel regime (*ε* > 0) the value of m approaches −1 and a solid-like response is detected. In the deficit of crosslinker agent added to the chitosan solution, no gel network is expected to evolve. However, when a sufficient amount of crosslinker agent is added to a semidilute chitosan solution, a macroscopic gel is formed, and it is shown above (Figure 3a) that the incipient gel is developed faster with increasing crosslinker concentration. Intuitively, the solid-like response (*m* = −1) after the gel point is expected to be approached faster when the crosslinker concentration is higher [50,51]. However, as can be seen from Figure 4a–c, this is not the case. For instance, *m* = −1 at *ε* = 1 at a GCA concentration of 0.25 wt.%, whereas for a concentration of 1.0 wt.% *m* = −0.84 for *ε* = 1 and *m* = −0.97 for *ε* = 2. This means that the distance from the gel point to the solid-like performance is longer for a higher than a lower crosslinker concentration. This finding is counterintuitive but may be related to the complex reaction scheme with different reaction paths as outlined in Supplementary Materials Figure S2. It seems that an excess of crosslinker agent inhibits the further crosslinking process after the gel point. This can probably be ascribed to competing reaction paths during the post gel stage. Factors that can affect the reaction rate are concentration of individual intermediates, solubility of components, stereo chemical issues, and kinetics.

The effect of crosslinker concentration on the time evolution of the power law exponent m for 1 wt.% chitosan solution is depicted in Figure 4d. From the low values of m (close to zero) in the pre-gel regions of the systems it is evident that the samples exhibit a liquid-like response. It should be noted that, even at the gel points, the values of m are quite low for all systems; suggesting that the incipient gels are quite soft. Solid-like gels (*m* ≅ −1) are found for all systems after long crosslinking times; this demonstrates that the crosslinking process continues for a long period of time after the gel point.

### 2.3. Effects of Temperature and pH on the Gelation Features 

**Figure 5:**
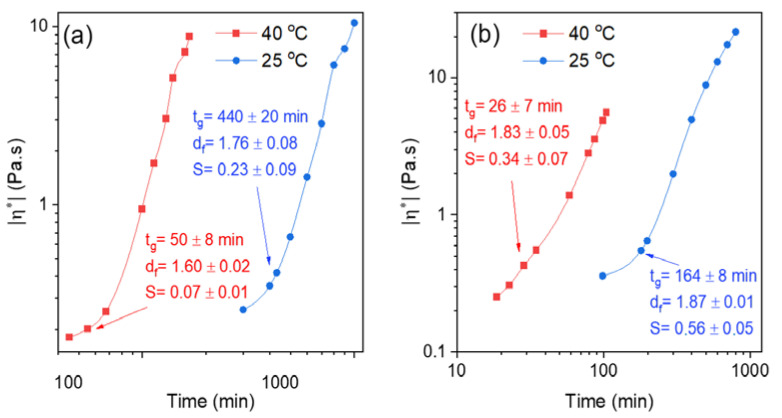
Time evolution of the absolute value of the complex viscosity during the gelation process of 1 wt.% chitosan at pH = 5.8 in the presence of 0.5 wt.% GCA (**a**) and 1 wt.% GCA (**b**) at the temperatures indicated. The values of the gel point (*t_g_*), fractal dimension (*d_f_*), and gel strength (*S*) for the incipient gels are given in the panels of the figure.

In this section, it will be shown how temperature and pH affect the gelation properties. Figure 5 shows the effect of temperature on the time evolution of the absolute value of the complex viscosity at two different crosslinker concentrations for 1 wt.% chitosan solutions at a pH of 5.8.

The behavior of the complex viscosity is similar at the two different crosslinker concentrations, but as discussed above the gelation process is faster at the higher crosslinker concentration. The effect of temperature on gelation is momentous and it is obvious that the gelation process accelerates at higher temperature. This finding is attributed to boosted mobility of the crosslinker molecules at elevated temperature, and the higher collision frequency between the active sites of the polymer and crosslinker molecules leads to faster gelation. This type of behavior has been reported in the literature for chemically gelling polymer systems of various natures [50,58,64,65,66]. At low crosslinker concentration (0.5 wt.%), increasing temperature seems to give a somewhat lower value of *d_f_*, suggesting a less-tight incipient gel network and a smaller value of S. Comparable effects are observed at the higher crosslinker concentration. These observations can probably be rationalized in terms of the higher mobility of the polymer chains at elevated temperatures, as this weakens the intermolecular connections of the polymer chains and the network becomes more “open”. Similar temperature effects on *d_f_* and *S* were reported for chemically crosslinked dextran gels [50]. 

**Figure 6:**
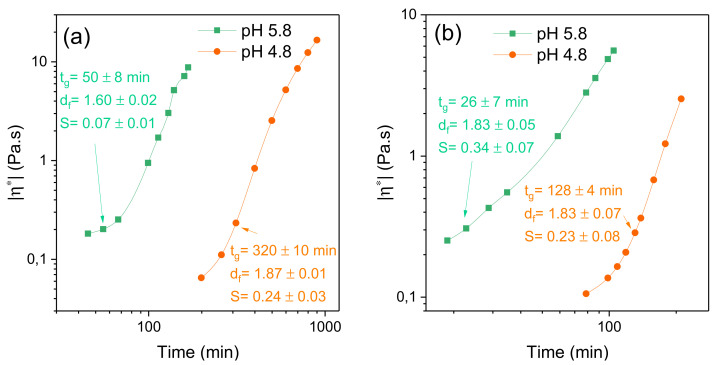
Time evolution of the absolute value of the complex viscosity during the gelation process of 1 wt.% chitosan at 40 °C in the presence of 0.5 wt.% GCA (**a**) and 1 wt.% GCA (**b**) at the pH values indicated. The values of the gel point (*t_g_*), fractal dimension (*d_f_*), and gel strength (*S*) for the incipient gels are displayed in the figure.

The free amino groups (-NH_2_) of chitosan play a critical role in the formation of crosslinked hydrogels (see Supplementary Information). At pH values below its pK_a_ (pH ≈ 6.3–7), the number of protonated amino groups (-${\mathrm{NH}}_{3}^{+}$) increases and chitosan becomes water-soluble [67,68,69]. The electrostatic repulsion between the polymer chains then leads to the swelling of the gel network. The intrinsic dissociation constant pK_0_ when the net charge goes to zero has been reported be pK_0_ = 6.5 [70]. The protonated amino groups are not participating in the crosslinking reaction; this suggests that the number of active sites for crosslinking is gradually as pH drops below the pK_a_ value. This effect is illustrated in Figure 6, where the time evolution of the absolute value of the complex viscosity during the gelation of 1 wt.% chitosan solutions in the presence of different amounts of GCA at pH values of 4.8 and 5.8 is depicted. The most conspicuous feature is the earlier advancement of the viscoelastic response and the much longer gelation time for the solutions with the lower pH value. The characteristic gelling features are similar at both crosslinker concentrations but, as discussed above, a higher crosslinker concentration expedites the gelation process. It is obvious that the small pH jump from 5.8 to 4.8 has a substantial impact on the gelation process. This is attributed to the reduction in the number of deprotonated amino groups available for the crosslinking of the network when the pH value drops. However, at the low GCA concentration (0.5 wt.%) the values of the fractal dimension at different pH would indicate a tighter incipient gel network at the lower pH; this seems to be counterintuitive, considering the lower number of free amino groups for crosslinking at low pH. At a higher GCA concentration (1 wt.%), the fractal dimension (*d_f_* = 1.8) is the same for both pH values. We have no explanation for the lower value of *d_f_* observed at pH 5.8 for the low GCA concentration.

### 2.4. Effect of GCA on the Mesh Size of Mature Gels

An important and characteristic parameter for the gel network is the mesh size or pore size that can be estimated from rheological experiments [71,72]. In the framework of rheological characterization and the classical theory of rubber-elasticity [73,74], the average mesh size of the gel network can be estimated from the storage modulus *G*′ at infinitesimal deformations. On the basis of this, the following relationship is employed

*G*′ = *nRT* (5)

where *n* is the number density of elastically effective crosslinking points (mol/m^3^), *R* is the ideal gas constant, and *T* is the absolute temperature. In view of this, at a given temperature, a rise in the value of *G*′ is correlated with a proportional increase in the number of network junctions. In the present work, it is assumed, for simplicity, that the gel-network contains crosslinking points that are evenly spread out and that each one is located in the center of a cubic-shaped volume element [50,51,75,76,77,78]. In this arrangement, the length *L* of a side of the cubic element can be determined because all cubic elements are combined to span the whole gel volume. The total number of junctions can then be calculated from Equation (6), where the pore “radius” in the network is *L*/2:

(6) $$L=\xi_{cub}={\left(\frac{1}{nN_{A}}\right)}^{1/3}={\left(\frac{RT}{G^{\prime }N_{A}}\right)}^{1/3}$$

where *N_A_* is Avogadro’s constant. Some other groups [79,80,81] have utilized another model, where the gel-network is pictured as consisting of an assembly of spherical elements, where the volume associated with each crosslink in the real network is that of a sphere centered in the crosslink and characterized by a diameter equal to the average mesh size (*ξ_sph_*). In this approach, the relation between the storage modulus and the average mesh size can be written as [70]:

(7) $$\;\xi_{sph}={\left(\frac{\pi }{6}\right)}^{1/3}{\left(\frac{RT}{G^{\prime }N_{A}}\right)}^{1/3}$$

The difference between the two models is small, *ξ_cub_* = 1.24 *ξ_sph_*, and our focus is not primarily on the absolute numerical values of the mesh size, but rather on the trends when the crosslinker concentration and temperature are changed. 

**Figure 7:**
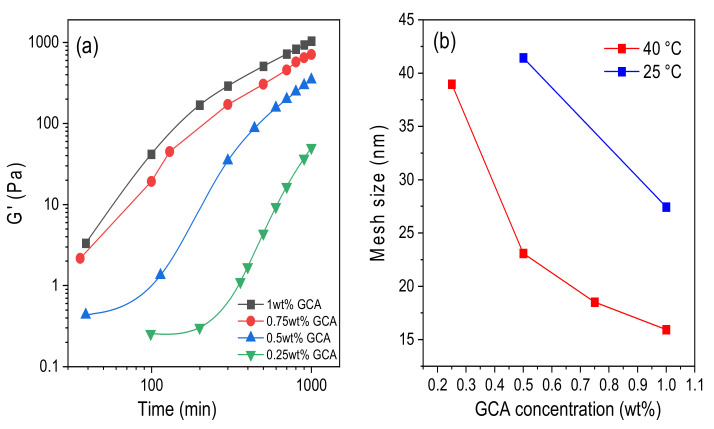
(**a**) Time evolution of the storage modulus at 40 °C, taken at a fixed low angular frequency (7 rad/s), during the gelation process at pH 5.8 and at the crosslinker concentrations indicated. (**b**) Effects of crosslinker concentration on the mesh size (calculated from Equation (6)) after a long curing time of 18 h at the temperatures indicated.

Figure 7a shows the time evolution of the storage modules at various crosslinker concentrations at 40 °C. A common feature is the strong rise of *G*′ with increasing curing time; the magnitude of this effect is strengthened with growing level of crosslinker addition. It is evident that both increasing crosslinker concentration and time of curing generate augmented crosslinking density and a more rigid and elastic network with higher values of *G*′.

By using the fractal concept in the analysis of incipient gels (see Figure 3c), it is concluded above that increasing crosslinker concentration led to tighter gel structure. It is interesting to note that, even after 18 h curing time, the mesh size of the gel continues to shrink as the crosslinker addition increases (Figure 7b). This suggests that there are still many active sites in the gel network to be crosslinked after the incipient gel has been formed. To be able to create mechanically stable gel networks as scaffolds in tissue engineering, one can play with both the curing time and the crosslinker concentration. It is well-established for various polymer/chemical crosslinker systems [50,51,76,81] that the pore size or mesh size shrinks with increasing crosslinker concentration. 

Furthermore, Figure 7b reveals a significant temperature effect on the pore size of the long-cured (18 h) gel network. It is clear that, at a fixed crosslinker concentration, an elevated temperature gives rise to a compaction of the network and a smaller average mesh size. The results at the gel point (cf. Figure 5) also demonstrate much faster gelation at the higher temperature, but in terms of the fractal dimension, the tightness of the gel structure seems to be virtually unaffected by temperature. It is not unreasonable that a long curing time at a high temperature may lead to a tighter network structure, due to the increased probability of a completed crosslinking reaction.

### 2.5. Comparison of Gel Formation of Chitosan with Different Crosslinker Agents

**Figure 8:**
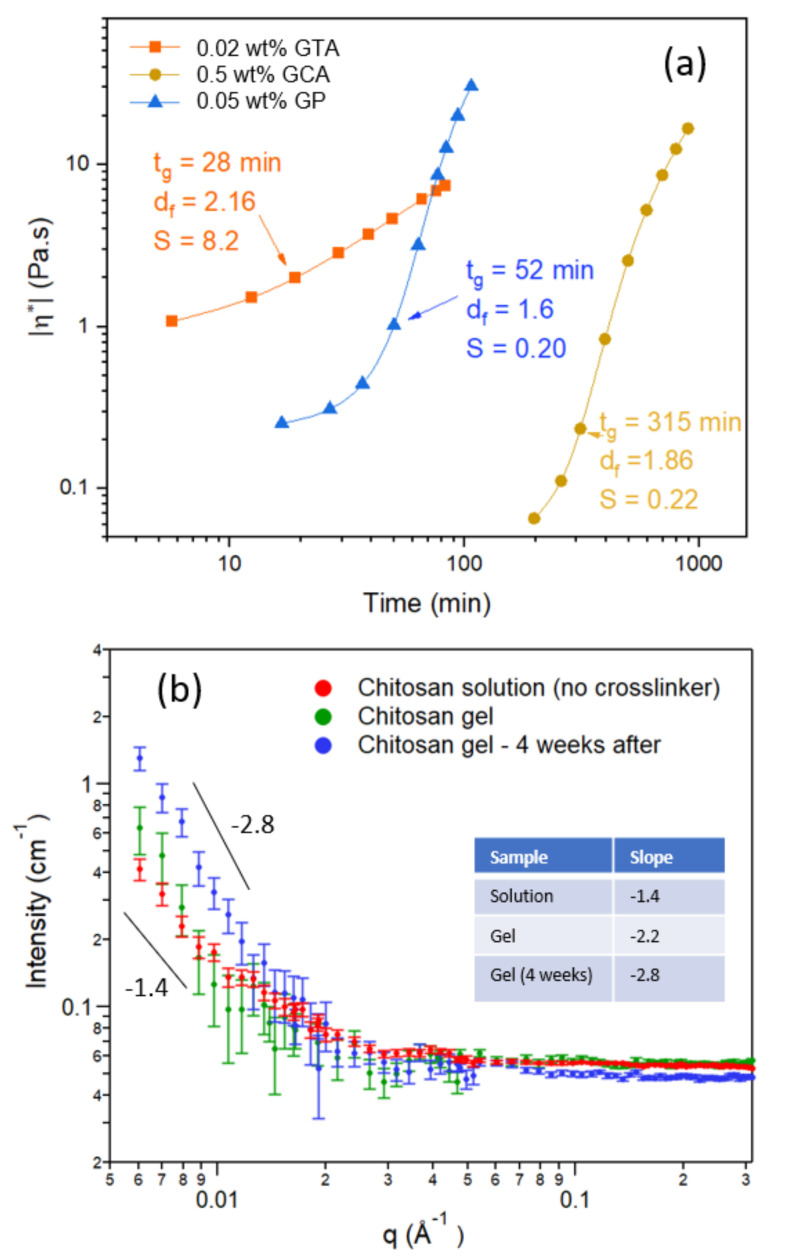
(**a**) Time evolution of the complex viscosity for 1 wt.% chitosan in the presence of the indicated crosslinker concentrations and different crosslinker agents: glutaraldehyde (GTA), glyceraldehyde (GCA), and genipin (GP) at pH 5 and 40 °C. (**b**) Small angle neutron scattering profiles in 1 wt.% chitosan samples without crosslinker and in the presence of 1 wt.% genipin for an incipient gel and a mature gel (4 weeks after gelation).

Figure 8a shows the time evolution of the absolute value of the complex viscosity during the crosslinking process of chitosan with different crosslinker agents (glutaraldehyde (GTA), glyceraldehyde (GCA), and genipin (GP)). GCA and GP are agents that are considered to exhibit low cytotoxicity, whereas GTA is a commonly used crosslinker that is not recommended for biomedical applications due to its higher cytotoxicity. The graphs display the time development of |η*| during gelation to mature gels. Several factors, such as the type of reaction mechanism for gelation, pH, and crosslinker concentration will affect the gelation process. Since the needed crosslinker concentration to induce the gelation of chitosan is different for the agents, the gelation mechanism of chitosan is dissimilar, depending on the type of crosslinker. In view of this, it is very difficult to attain matching conditions with the different crosslinker agents so that the characteristic gelation features for the corresponding gels can be compared in an unambiguous manner. It has been shown that the gelation mechanism of chitosan is different when GTA [7], GCA [16], or GP [12,15] are employed as crosslinker agents. 

It is evident from Figure 8a that the overall viscosification rate of chitosan in the presence of 0.02 wt.% GTA is rather slow compared with those obtained with GP and GCA, but the gelation time is short, and the gel strength is high compared with the other crosslinker agents. This suggests that, in the presence of GTA, strong incipient gels are formed with a tight network structure. The gelation of chitosan with GTA requires only a low crosslinker concentration and somewhat higher (0.05 wt.%) for GP, whereas with GCA a fairly high concentration is required at this pH (pH 5). The gel strength is practically the same for GP and GCA with a more open network structure in the presence of GP.

Figure 8b shows small angle neutron scattering (SANS) results for a 1 wt.% solution of chitosan, incipient gel, and a matured gel with GP as the crosslinker agent. An inspection of the results reveals that in the low wave vector (*q*) range the scattering profile is changed. The slope for 1 wt.% chitosan solution without GP is close to −1.4 and this is typical for solutions containing extended coil-like polymer chains. When an incipient gel is formed, we observe a higher value of the slope (−2.2) suggesting local compaction of the network. After four weeks of curing of the gel a slope of −2.8 is observed and the gel-network is further compacted. This is compatible with the presented rheological results for the time evolution of mature gels in the presence of GCA. 

## 3. Conclusions

In this work, the gelation of chitosan solutions in the presence of the non-cytotoxic chemical crosslinker agent glyceraldehyde (GCA) is characterized by rheological methods. The findings demonstrate that a systematic rheological classification of the gelation process can be conducted, both in the pre-gel and the post-gel stages by using GCA. The results support our hypothesis that the biocompatible GCA constitutes an attractive crosslinker agent in forming tunable chitosan hydrogels that can be systematically characterized by rheological methods.

The entanglement concentration (*c_e_*) was found to be ≈0.2 wt.% and the power laws for the zero-shear specific viscosity below the entanglement concentration could be described as $\eta_{sp}^{0}$~*c^α^* with *α* ≈ 1 and above *c_e_* as $\eta_{sp}^{0}$~*c^β^* with *β* ≈ 3.2, and with virtually no pH or temperature effect.

**Figure 9:**
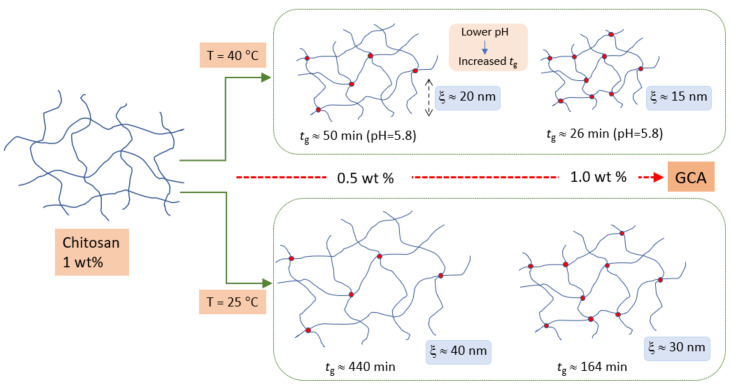
Schematic illustration of the GCA-crosslinked chitosan system and how it is influenced by temperature, GCA concentration, and pH. Increasing the temperature (from 25 °C to 40 °C) promotes faster gelation and a tighter network (represented by the parameter *ξ*). An increase in the GCA concentration leads to a faster gelation and a tighter network, both at low and high temperature, while lowering the pH has the effect of a significant increase in the gelation time.

In the formation of incipient chitosan gels, the results clearly show that the gelation time decreases with increasing values of the crosslinker concentration, temperature, and pH. A tighter gel-network develops with increasing crosslinker concentration, whereas changes in temperature and pH have a more modest influence on the tightness of the network. In addition, the gel strength rises with increasing GCA concentration. A schematic illustration of the effects of crosslinker concentration and temperature on the gel-structure is depicted in Figure 9.

The frequency dependency of the absolute value of the complex viscosity can be described by a power law (|η*(*ω*)*|*~*ω^m^*) and the substantial change in the exponent m after the gelation point shows that pronounced crosslinking occurs over a long time in the post-gel region. The transition from liquid-like to solid-like behavior accelerates when the crosslinker concentration increases. In addition, higher temperature and pH values expedite this transition. After a long curing time (18 h) of the gel, the porosity of the gel network decreases with increasing crosslinker concentration. The decrease is much stronger at a high temperature. Overall, the results from this study reveal that the crosslinking process of the gel is favored by high crosslinker concentration, elevated temperature, and pH values close to the pK_a_ value for chitosan.

## 4. Materials and Methods

### 4.1. Materials and Preparation of Gels

In all experiments, MilliQ water was used. The chitosan sample, designated Chitopharm^®^L, was given as a gift from Chitinor AS, Tromsoe, Norway and it has a degree of deacetylation of 87.4% and a weight average molecular weight *M_w_* of Ca. 700 kDa, and a dispersity index (*M_w_*/*M_n_*) of 2.3. DL-glyceraldehyde 90% was obtained from Sigma Aldrich, Oslo, Norway. Glacial acetic acid and sodium hydroxide were both purchased from Merck. A chitosan stock solution was prepared by dissolving chitosan in 1 vol% acetic acid and a magnetic stirrer was used to homogenize the solution at ambient temperature for 10–12 h. The pH of the solution was adjusted to the prescribed values by adding aqueous 10 M NaOH dropwise to the chitosan solution. The pH was measured by utilizing a Mettler Toledo™ FE20 FiveEasy™ benchtop pH meter. In the crosslinker reactions, glyceraldehyde was dissolved in MilliQ water and the agent was added dropwise under magnetic stirring to the chitosan solution to obtain the prescribed concentration of chitosan (fixed concentration of 1 wt.%) and the crosslinker concentrations (from 0.25 wt.% to 1 wt.%) and pH was adjusted to the prescribed values. After 2 min of stirring, the reaction mixture was poured onto the rheometer plate and the rheology experiments were commenced. In terms of the ratio *r* = weight% GCA/weight% chitosan, r assumes values between 0.25 to 1.0. The viscosities of the solvent (1 vol% acetic acid) at 25 °C and 40 °C were found to be 0.915 mPas and 0.674 mPas, respectively.

### 4.2. Rheology Experiments

Oscillatory shear and shear viscosity measurements of the samples were carried out on a rheometer (Physica MCR 301, Anton Paar, Graz, Austria) employing a cone-and-plate geometry, with a cone angle of 1° and a diameter of 75 mm, for all the experiments. The samples were put onto the plate, and to prevent evaporation of the solvent, the free surface of the sample was always covered with a thin layer of a low-viscosity silicone oil (the value of the viscosity is practically not affected by this layer) [50,51]. The measuring device is equipped with a temperature element (Peltier plate) that promotes an efficient temperature control (±0.05 °C) over an extended time for the temperatures (25 °C and 40 °C) considered in this work. The values of the strain amplitude were checked to ensure that all measurements were conducted within the linear viscoelastic regime, where the dynamic storage modulus (*G*′) and loss modulus (*G*″) are independent of the strain amplitude. Stress sweep experiments were carried out to observe the linear viscoelastic regime. The stress sweep measurements were performed in the strain range from 0.01 to 50% at fixed angular frequencies of 0.1, 1, 5, and 10 rad/s (see Supplementary Materials Figure S3). It is shown that the storage modulus is independent of strain in the considered domain for the experimentally relevant angular frequencies.

Viscosity measurements to determine the intrinsic viscosity were performed with a standard Ostwald viscometer, placed into a temperature-controlled water bath.

### 4.3. Small Angle Neutron Scatering (SANS) Experiments 

Neutron scattering experiments were carried out using the SANS instrument at the JEEP II reactor at the Institute for Energy and Technology (IFE) at Kjeller, Akershus, Norway. A velocity selector (Daimler-Benz Aerospace Dornier, Friedrichshafen, Germany) was employed with a wavelength spread of Δ*λ*/*λ* = 10%. Two different sample detector distances (1.0 m and 3.4 m) and two different neutron wavelengths (5.1 Å and 10.2 Å) were used to obtain a total scattering range (*q*-range) from 0.006 Å*^−^*^1^ to 0.32 Å*^−^*^1^, where *q* is defined by *q* = (4π/*λ*)sin(*θ*/2), with *θ* being the scattering angle and *λ* the neutron wavelength. The measurements were carried out in 5 mm cuvettes. Accurate temperature control was achieved by placing the sample cell onto a copper base with internal water circulation. The normalized scattering intensity, i.e., the absolute scattering cross section (cm^−1^), was calculated by incorporating the contribution from the blocked-beam background and the empty cell, including independent measurements of the transmissions. In order to reduce incoherent background and enhance the contrast, the samples were prepared in heavy water.

## Data Availability

Data can be obtained from the authors upon request.

## Supplementary Materials

The following materials are available online at www.mdpi.com/article/10.3390/gels7040186/s1, Figure S1: Shear-rate dependence of the viscosity of chitosan solutions of various concentrations at pH 5 and at a temperature of 25 °C. Figure S2: Schematic illustration of various steps in the crosslinking reaction of chitosan with glyceraldehyde and above this scheme, a brief discussion of the reaction paths is given. Figure S3: Stress sweep experiments were carried out to observe the linear viscoelastic regime and all experiments were performed in this regime. It was shown that the storage modulus is independent of strain in the considered domain for the experimentally relevant angular frequencies.
