# Supplementary material for: Glyceraldehyde as an Efficient Chemical Crosslinker Agent for the Formation of Chitosan Hydrogels
Source: Gels. 2021 Oct 28;7(4):186. doi: 10.3390/gels7040186 (PMC8628775; doi:10.3390/gels7040186)
Supplement: Supplementary file 1 [file gels-07-00186-s001.zip › gels-1413450-supplementary.pdf]

Supplementary Information:

Glyceraldehyde as an Efficient Chemical Crosslinker Agent for the Formation of Chitosan Hydrogels

Pierre Carmona[1,2], Anca M. Tasici[3], Sverre A. Sande[3], Kenneth D. Knudsen[4] and Bo Nyström[1,*]

1 Department of Chemistry, University of Oslo, P.O. Box 1033, Blindern, N-0315 Oslo, Norway; pierre.carmona@chalmers.se (P.C.); bo.nystrom@kjemi.uio.no (B.N.)
2 Unit Product Design, Department Agriculture and Food, Division Bioeconomy and Health, RISE Research Institute of Sweden, Frans Perssons väg 6, Box 5401, SE-402 29, Gothenburg, Sweden; Division Nano-and BioPhysics, Department of Physics, Chalmers University of Technology, Fysikgränd 3, 412 96, Gothenburg, Sweden
3 School of Pharmacy, Department of Pharmaceutics, University of Oslo, P.O. Box 1068, Blindern, N-0316 Oslo, Norway; ancamtasici90@gmail.com (A.M.T.); s.a.sande@farmasi.uio.no (S.A.S.)
4 Institute for Energy Technology, P. O. Box 40, N-2027 Kjeller, Norway; Kenneth.knudsen@ife.no
* Correspondence: bo.nystrom@kjemi.uio.no; Tel.: +47 22855522

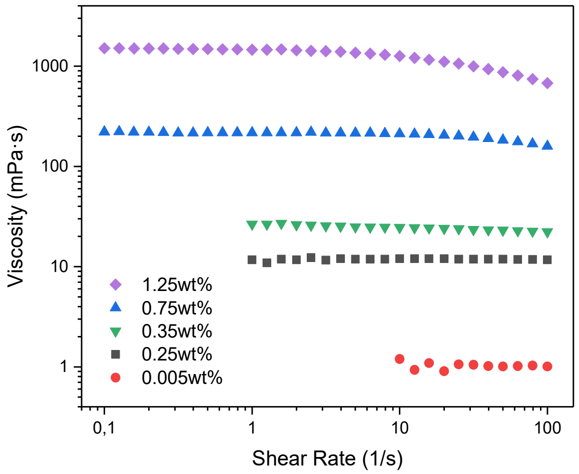

Figure S1. Shear-rate dependence of the viscosity for the indicated chitosan concentrations at pH 5 and at a temperature of 25 °C.

Crosslinking of chitosan with DL-glyceraldehyde.

The Maillard reaction encompasses a complex series of non-enzymatic reactions that are initiated by the condensation of an amine (e.g., from amino acids, peptides, and proteins) with a carbonyl compound, usually a reduced sugar (e.g., glyceraldehyde). There are many studies in the literature dealing with crosslinking reactions of proteins via the Maillard reaction [1-4]. However, the polymer chitosan contains free amino groups that can be crosslinked in a similar way to produce a chitosan gel-network. An outline of the reaction between amino groups and DL glyceraldehyde (GCA) was suggested by Tessier et al. [5] on the basis of this approach; a schematic illustration of the crosslinking of chitosan is presented in Figure S2. As a Maillard reaction, the aldehyde group of GCA first undergoes a nucleophilic attack (S_N2) by the primary amino group of chitosan, forming an intermediate Schiff base between GCA and the amine group of chitosan. The relative unstable Schiff base undergoes rearrangement to form a more stable Amadori product, which goes through a series of further reactions through dicarbonyl intermediates to form advanced glycation end products.

In pathway A, the tautomerization results in the formation of an aldehyde group. This aldehyde group can react with another chain of chitosan as before (A1) or polymerize with other GCA molecules to give an aldol condensation (A2). In pathway B, the secondary amino group reacts with an aldehyde group of glyceraldehyde under a nucleophilic attack. This facilitates the GCA chains to react with other amino groups from chitosan chains (B1). Another possibility is the intramolecular aldol condensation (B2).

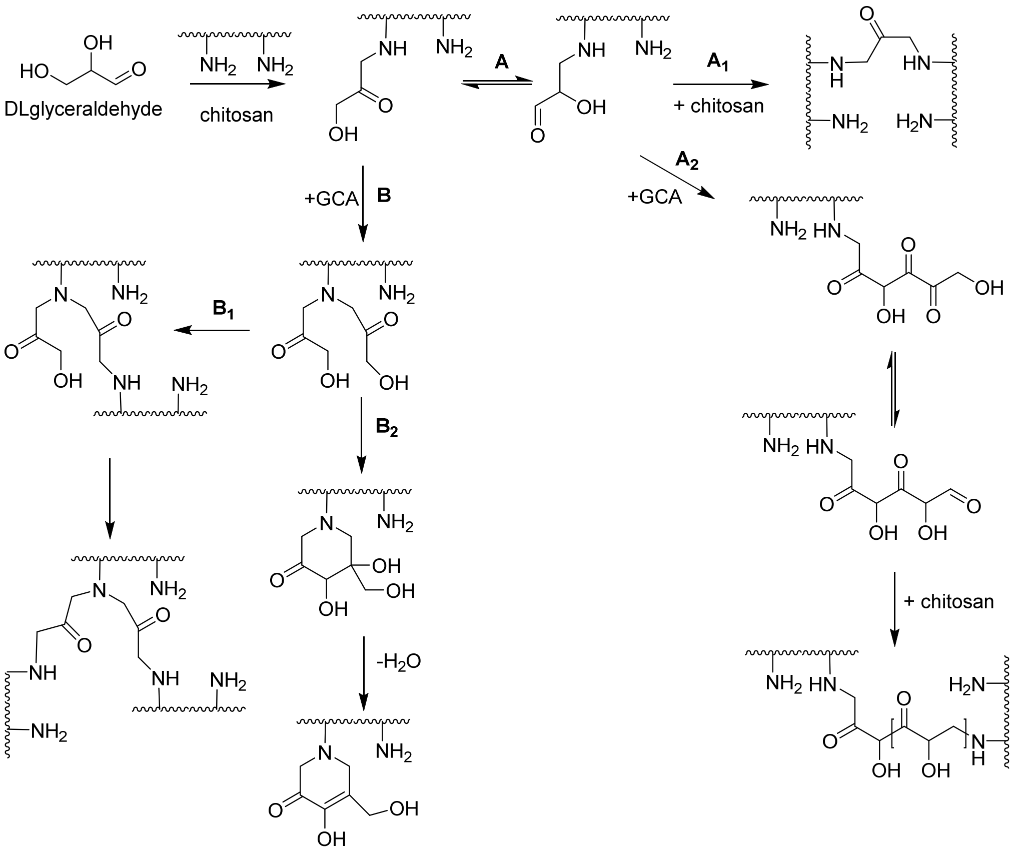

Figure S2. Suggested scheme for cross-linking of chitosan with GCA.

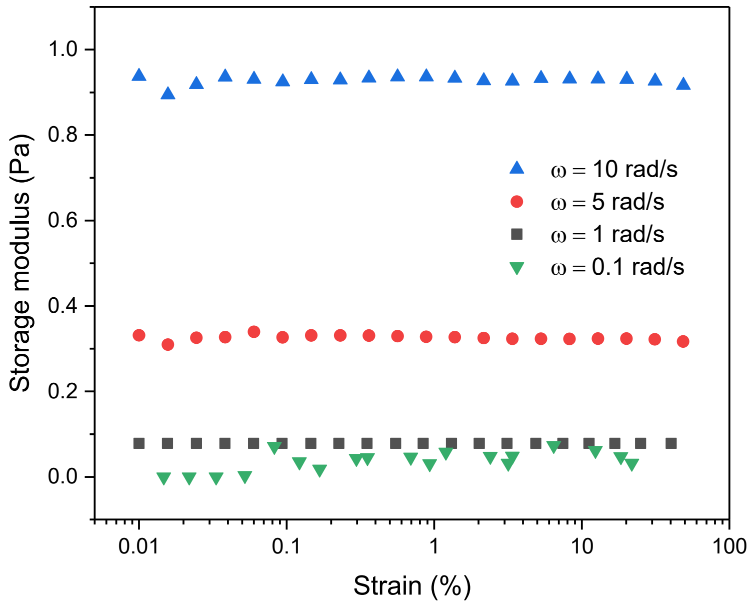

Figure S3. Check of the linear viscoelastic regime for 1wt% chitosan solutions at pH5.8. Plot of the storage modulus versus strain.

References

1. Al-Abed, Y.; Bucala, R. Structure of a synthetic glucose derived advanced glycation end product that is immunologically cross-reactive with its naturally occurring counterparts. *Bioconjugate Chemistry* **2000**, *11*, 39-45.
2. Glomb, M.A.; Lang, G. Isolation and characterization of glyoxal-arginine modifications. *Journal of Agriculture and Food Chemistry* **2001**, *49,* 1493-1501.
3. Biemel, K.M.; Reihl, O.; Conrad, J.; Lederer, M.O. Formation pathways for lysine- arginine cross-links derived from hexoses and pentoses by Maillard processes: unraveling the structure of a pentosidine precursor. *Journal of Biological Chemistry* **2001**, *276*, 23405-23412.
4. Ahmed, N.; Argirov, O.K.; Minhas, H.S.; Cordeiro, C.A.A.; Thornalley, P.J. Assay of advanced glycation endproducts (AGEs): surveying AGEs by chromatographic assay with derivatization by 6-aminoquinolyl-N-hydroxysuccinimidyl-carbamate and application to N∊-carboxymethyl-lysine- and N∊-(1-carboxyethyl)lysine-modified albumin. *Biochemical Journal* **2002**, *364*, 1-14.
5. Tessier, F.J.; Monnier, V.M.; Sayre, L.M.; Kornfield, J.A. Triosidines: novel Maillard reaction products and cross-links from the reaction of triose sugars with lysine and arginine residues, *Biochemical Journal* **2003**, *369*, 705-719.
